# An optical study on the enhanced light trapping performance of the perovskite solar cell using nanocone structure

**DOI:** 10.1038/s41598-024-56424-4

**Published:** 2024-06-11

**Authors:** Xiaowei Gu, Zeyu Li, Rusli E, Xiaoxiao Xu, Zhi Tao, Jiangyong Pan, Xuechao Yu, Linwei Yu, Sudha Mokkapati

**Affiliations:** 1 School of Electronic and Information Engineering, Nanjing University of Information Science and Engineering, Nanjing, 210044 China; 2 School of Electronic Science and Engineering, Nanjing University, Nanjing, 210093 China; 3 School of Electrical and Electronic Engineering, Nanyang Technological University, Singapore, 639798 Singapore; 4 Key Laboratory of Multifunctional Nanomaterials and Smart Systems, Suzhou Institute of Nano-Tech and Nano-Bionics, Chinese Academy of Science, Suzhou, 215123 China; 5 Department of Materials Science and Engineering, Monash University, Clayton, VIC 3800 Australia

**Keywords:** Optical physics, Energy science and technology, Energy harvesting, Energy storage, Renewable energy

## Abstract

Photon management strategies are crucial to improve the efficiency of perovskite thin film (PTF) solar cell. In this work, a nano-cone (NC) based 2D photonic nanostructure is designed and simulated aiming at achieve superior light trapping performance by introducing strong light scattering and interferences within perovskite active layer. Compared to the planar PTF solar cell, the NC nanostructured device with 45 degrees half apex angle obtains highest short-circuit current density, which improved over 20% from 15.00 mA/cm^2^ to 18.09 mA/cm^2^. This work offers an alternative design towards effective light trapping performance using 2D photonic nanostructure for PTF solar cell and could potentially be adopted as the nano-structuring strategy for the future perovskite solar cell industry.

## Introduction

Over the last decade, organic–inorganic perovskite thin film (PTF) solar cells have emerged as a promising candidate for next generation photovoltaic (PV) device due to its superior properties including long carrier diffusion length, high absorption coefficients, low defect density, lightweight and mechanical flexibility, etc.^1^. Despite its record high power conversion efficiency (PCE) of over 25%^2,3^, PTF solar cell’s efficiency would downgrade significantly at an active layer thickness of 300 nm or thinner, due to poor absorption for wavelength greater than 500 nm, which is close to its bandgap^4^. This limits its performance at scenarios where extra flexibility^5^, high power to weight ratio^6^ or lower material cost^7^ are pursued. Moreover, Wang, et al. estimated that more than 30% of the incident light were not collected by perovskite active layer due to reflection, transmission and parasitic absorptions^8^. Hence, there is great need and potential to enhance perovskite material’s light absorption by adopting photon management strategies^9^. Typical methods include surface plasmon resonance^10^, up/down conversion^11,12^, anti-reflection coating^13^, etc.

Among the various approaches, texturing PTF by using a well-designed periodically nanostructured substrate is proven to be effective in improving the light-trapping performance. Earlier works adopted robust but complex techniques like e-beam lithography and laser direct writing to create patterns on the substrates or on the perovskite film itself^14,15^. With the development of the low cost and large-scale compatible nanoimprint process^16^, which was later recognized to be a more prospective technique due to its merits including mass production compatibility and lesser process steps^17,18^, fewer surface defects^19^, high enhancement factors^16^, etc., perovskite solar cell (PSC) photon management by photonic nanostructure attracted further attentions. Recently, Song’s group developed a simple and elegant strategy to incorporate a 1D photonic grating structure^20^ and dual grating formed Moiré structure^21^ into the perovskite surface by using a commercially available CD/DVD discs. Light trapping by diffraction was proven to be effective, leading to an improved short-circuit current density (*J*_*sc*_) and PCE. Other than 1D grating structures, 2D photonic structures also received attentions due to its favorable performance for PSCs having a thickness below 300 nm^22^. Compared to other 2D photonic structures like nanowires and nanopillars, where bending stress tends to accumulate at the joint of the nanowire and the film below, which leads to easy breaking^23^, nanocones (NCs) are supported with a larger base and hence better mechanical stability, and are more suitable for flexible or stretchable devices^24^. Optically, this structure provides a smooth change in the effective refractive index facing the light incident direction, which would also be an added benefit to light trapping performance by reflection suppression^25^. Furthermore, several computational studies had successfully revealed great potential of various kinds of PTF SCs on their high performing PCEs^26–31^.

In this work, we design a 2D photonic structured NC based PTFs of only 200 nm thick and systematically investigated its light trapping performance by theoretical simulation. We firstly justify our simulation methodology by benchmarking our simulated results to experimental results from literature. After that, we compare the light trapping performance of perovskite NCs with different apex angles in terms of their absorptance spectra. Last but not lease, full devices with optimized geometries are constructed in the model, and their photovoltaic parametric are investigated. We found that perovskite NCs with 45 degrees half apex angle performs the best. Its *J*_*sc*_ performance was equivalent to a 300 nm thick planar device, while the material used was the same as a 200 nm thick planar device.

Our model suggested that a NC based PSC demonstrates effective light trapping performance. This approach is innovative because it leverages the unique optical properties of nanostructures to enhance light absorption in solar cells, provides insights into how light manipulation at the nanoscale can significantly enhance solar cell performance, as well as offers potential mechanical stability comparing to conventional nanowire based approaches. Overall, we successfully shown an alternative photon management design for future mass production commercial perovskite solar panels.

## Simulation method and benchmark

### Simulation parametric and methodology

**Figure 1:**
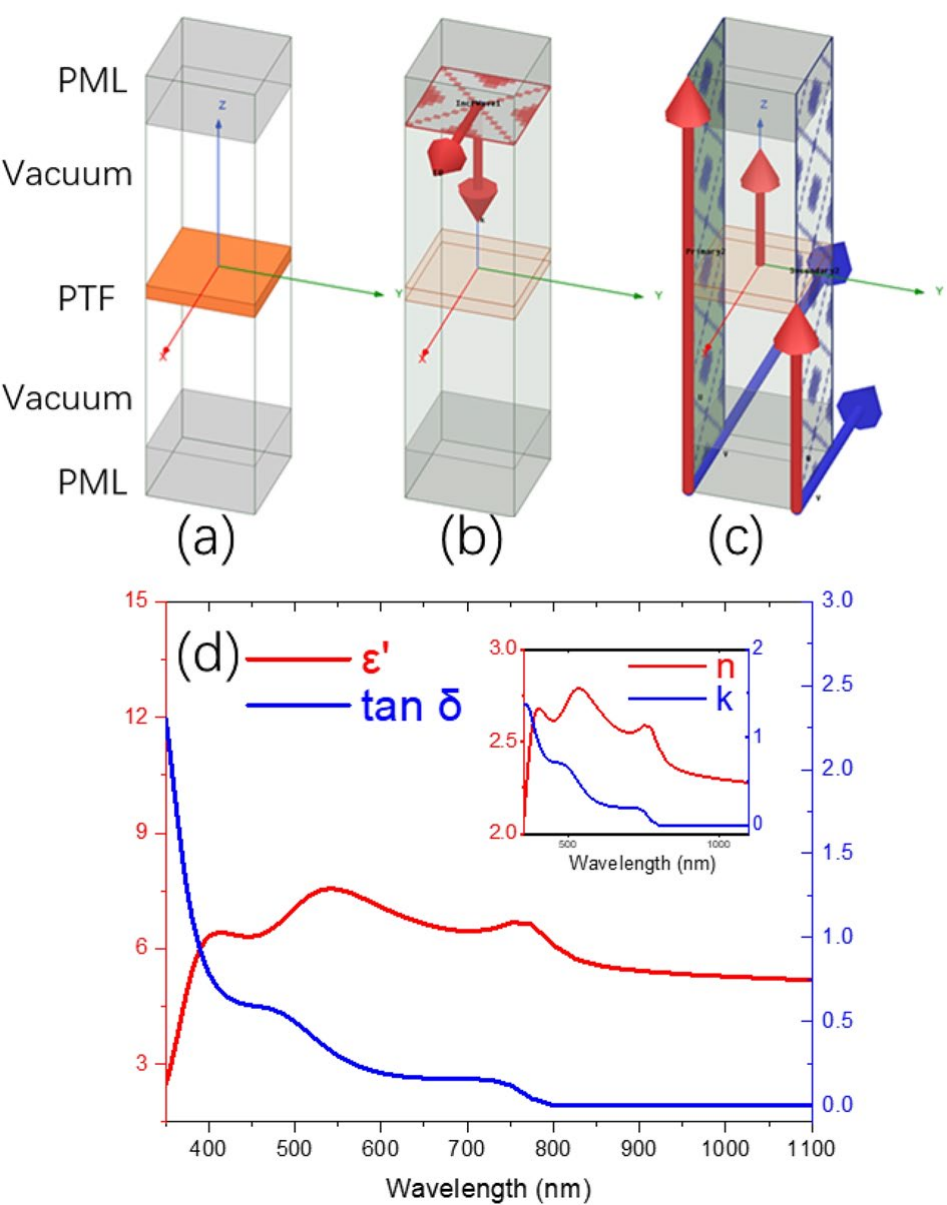
(**a**–**c**) Simulation model of a 300 nm thick planar PTF; (**d**) real part of the complex relative permittivity *ε*^′^, and dielectric loss tangent *tanδ* of the PTF used in model, inset shows the adopted *n* and *k* values, reprinted (adapted) with permission from^35^. Copyright 2015 American Chemical Society.

Figure 1a–c shows the details of the simulated unit cell of a 300 nm thick PTF in Ansys High Frequency Structure Simulator (ANSYS-HFSS) software. Figure 1a shows the different layers which are the top perfect matching layer (PML), top vacuum, PTF, bottom vacuum and bottom PML. Effect of the glass substrate were not considered due to a lower refractive index of glass than PTF, hence we approximated the glass substrate to vacuum. Electromagnetic (EM) plane wave excitation source was placed at the top of the unit cell, with electric field aligned to + x axis and propagation along –z axis, as illustrated in Fig. 1b. The frequency of the EM plane wave was swept from 858 to 270 THz with a step of 4 THz which corresponds to a wavelength range from 349.4 to 1110.3 nm, suited for PV characteristics investigation. Due to symmetry, deviation between TM and TE modes were not addressed^32^. In Fig. 1c, 2 pairs of master–slave surfaces were placed on the unit cell, which would set up the boundary condition with a continuous spatial EM values at opposite sides of the unit cell. Hence, the simulation of an infinite large PTF in vacuum could be realized. The simulated final results would be comparable with experimental data, considering light spot size would be much smaller than the sample size in any common spectrometer equipment. To investigate the optical performance of PTFs, the real part of the complex relative permittivity *ε*^*’*^, and dielectric loss tangent *tanδ* of the perovskite were calculated from the refractive index *n* and extinction coefficient *k* using Eq. (1)^33,34^, as shown in Fig. 1d. Table 1 shows the detailed input *n k*, values of the PTF as a function of wavelength.

1 $$\varepsilon^{\prime} = n^{2} - k^{2} ,\tan \delta = \frac{2nk}{{n^{2} - k^{2} }}$$

**Table 1:** Input n k, values of the PTF as a function of wavelength.

Wavelength (nm)	Refractive index *n*	Extinction coefficient *k*	Wavelength (nm)	Refractive index *n*	Extinction coefficient *k*
349.27	2.07	1.36	539.10	2.79	0.45
354.26	2.17	1.38	551.08	2.77	0.40
359.40	2.28	1.38	563.60	2.75	0.35
364.68	2.38	1.35	576.71	2.72	0.31
370.13	2.48	1.30	590.44	2.69	0.27
375.74	2.56	1.23	604.84	2.66	0.25
381.52	2.61	1.15	619.96	2.63	0.23
387.48	2.65	1.07	635.86	2.61	0.22
393.63	2.67	1.00	652.59	2.58	0.21
399.98	2.68	0.93	670.23	2.56	0.20
406.53	2.67	0.87	688.85	2.55	0.20
413.31	2.66	0.82	708.53	2.55	0.20
420.31	2.65	0.78	729.37	2.56	0.19
427.56	2.63	0.76	751.47	2.59	0.15
435.06	2.62	0.74	774.95	2.58	0.05
442.83	2.62	0.73	799.95	2.47	0.00
450.88	2.61	0.72	826.62	2.39	0.00
459.23	2.62	0.71	855.12	2.36	0.00
467.90	2.63	0.71	885.66	2.34	0.00
476.89	2.66	0.70	918.46	2.32	0.00
486.25	2.69	0.68	953.79	2.31	0.00
495.97	2.72	0.66	991.94	2.30	0.00
506.09	2.75	0.62	1033.27	2.29	0.00
516.64	2.77	0.57	1078.20	2.28	0.00
527.63	2.79	0.51	1127.21	2.27	0.00

### Planar and nanostructured thin film benchmark

**Figure 2:**
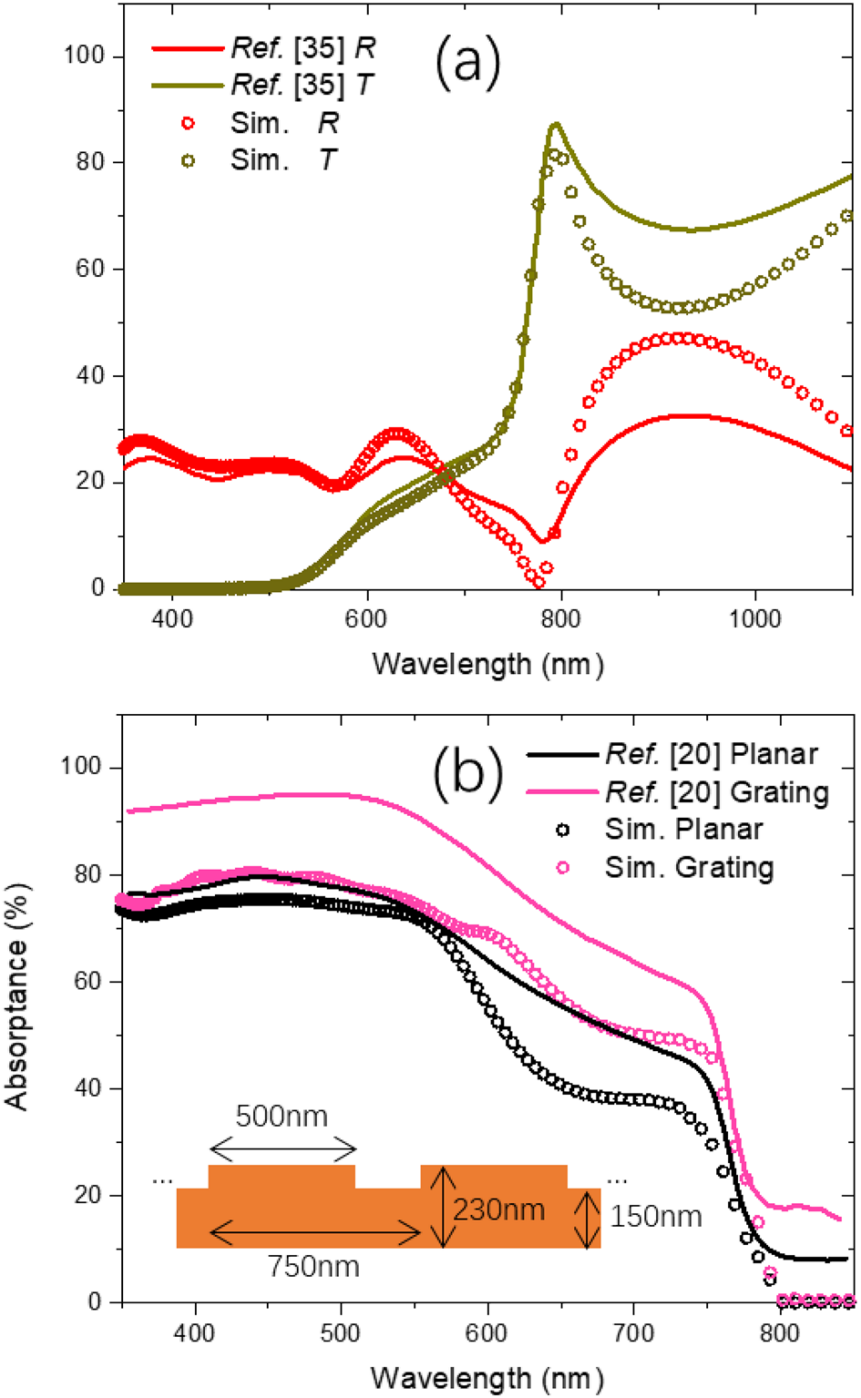
(**a**) Comparison of the *R* and *T* between our simulated 300 nm thick planar PTF and reference^35^ experimental results; (**b**) simulated absorptance from 350 to 850 nm of a 200 nm thick planar and diffraction grating structured PTF.

To benchmark our simulation method, we firstly simulated a 300 nm thick planar PTF. Reflectance (*R*) and Transmittance (*T*) results were analyzed and compared with reference^35^ experimental results, as shown in Fig. 2a. Regardless that our simulated *RT* results agree reasonably well with the reference, our model slightly over estimated the *R* in the 350–400 nm and 590–670 nm ranges. This might be due to at the surface of the real PTF sample, the nano-scale roughness would introduce extra scattering which lead to lower *R*^36^, while the simulation model considers only flat surfaces^37^. From 700 to 800 nm, an underestimation of *R* was observed. This might also be due to the nano-scale roughness, as it would induce extra forward scattering of the incident light, reducing the amount of light that enters the thin film^4,38^. Additionally, a destructive interference would occur following Eq. (2) in the above wavelength range, leading to all light entered the thin film being transmitted. As a result, an underestimation of the simulated *R* was observed.

2 $$2 \times n_{film} \left( \lambda \right) \times d_{film} \times \cos \left( {\theta_{incident} } \right) = m \times \lambda$$

From Fig. 1d inset, the PTF’s *n*_*film*_*(750 nm)* is 2.56, while the film thickness *d*_*film*_ is 300 nm as in the planar design as seen in Fig. 1a, and the resultant *m* is ~ 2.05, which fulfilled the destructive interference condition at the normal incident where *θ*_*incident*_ is 0°^37^. For wavelength from 800 nm onwards, significant underestimation (> 25%) was observed, which might due to sub band gap defects in PTF leading to undesired absorption^39^. Since no photocurrent would be generated beyond the bandgap of perovskite, light trapping of longer wavelength than 800 nm would not be considered. Hence such mismatch from 800 nm onwards can be ignored as of this work’s concern.

To further validating the correctness of our method under the nanostructured scenario, we investigated a DVD imprinted light diffraction grating structure of a nanostructured PTF with reference to^20^. Figure 2b shows both our and reference’s simulated absorptance from 350 to 850 nm of a 200 nm thick planar and diffraction grating structured PTF. Our simulated absorptance (*A*) was calculated from *A%* = *100%-R%-T%*^40,41^. Inset shows the side view and dimension of the diffraction grating, similar to the PTF geometry designed in^20^. Consistently, significant absorptance improvement was also observed in our results. Since absorptance enhancement would improve the power conversion efficiency (PCE) of the PV device due to the higher photocurrent generated arising from the more efficient light collection proven by^20^, absorptance spectra could act as a qualitative metrics to characterize the light trapping capability of the nanostructures.

## Results and discussion

### Light trapping properties of PTF NCs

**Figure 3:**
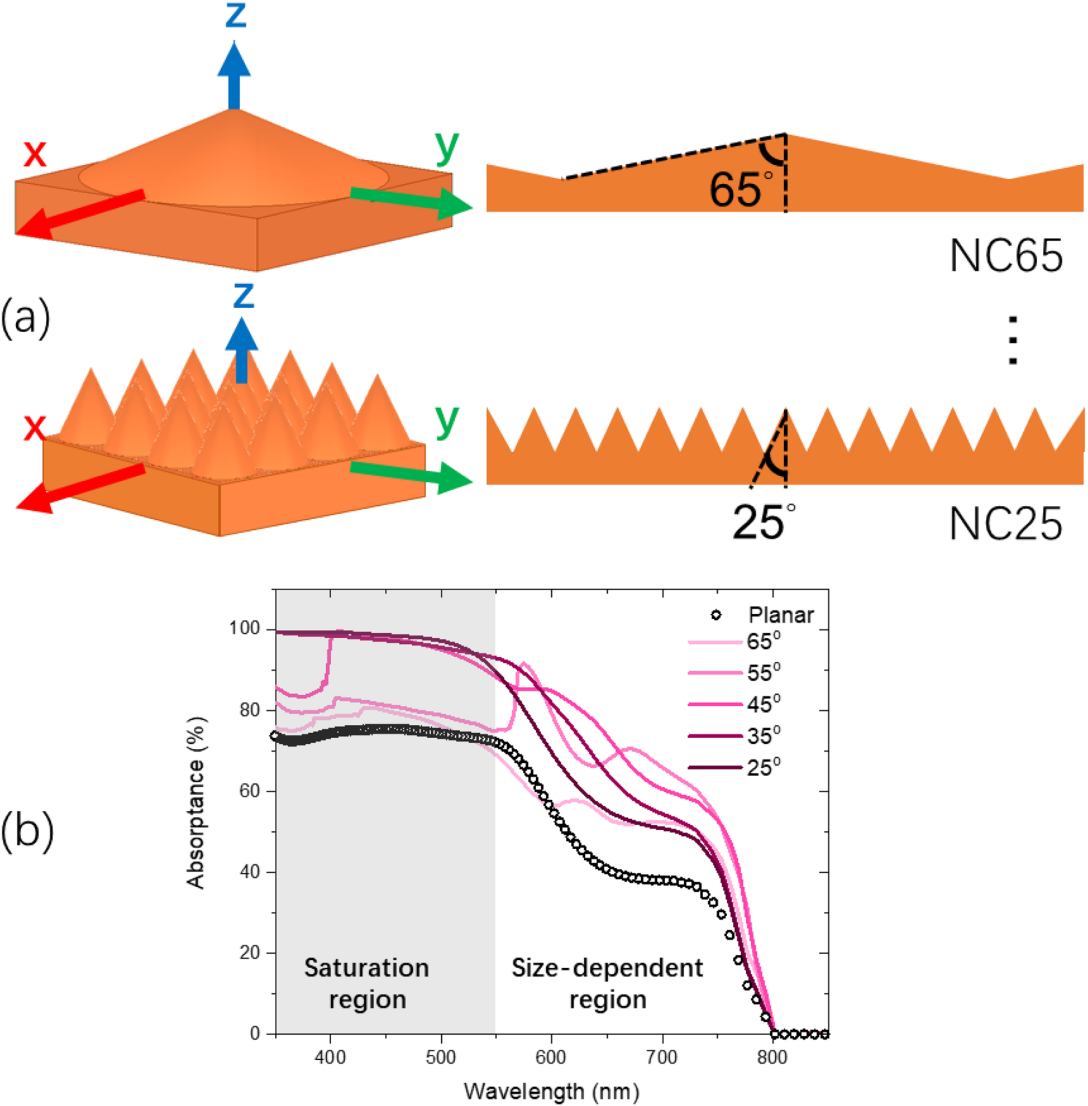
(**a**) The 3D schematic of NC65 and NC25 PTFs; (**b**) simulated absorptance results for different PTFs.

In the first part of the results and discussion, due to previously mentioned merits associated with cone based nanostructures, we investigated the light trapping properties of PTF NC with different apex angle designs, i.e. the sharpness of the structure. We fixed the cone height to be 200 nm and bottom PTF thickness to be 150 nm. This would ensure the same material volume per unit area comparing to both 200 nm thick planar and DVD grating PTFs in the last part. Figure 3a shows the 3D schematic of the unit cells and the side view along the zy plane of half apex angle set at 65 and 25 degrees, referring to as NC65 and NC25 PTFs respectively. The NC base radius increases with increasing half apex angle and the planar FTF could be considered as NC90 with infinite base radius. Figure 3b shows the absorptance results for planar, NC25, NC35, NC45, NC55 and NC65 PTFs. Significant absorption enhancement of all NC structured PFT was observed. We also saw two distinctive regions with different enhancement mechanism: (1) for *λ* < 550 nm, the enhancement saturates for half apex angle smaller than 45 degrees, which we referred to as “Saturation region” and (2) for *λ* > 550 nm, an optimized enhancement effect can be clearly observed for NC45 and NC55 PTFs, which we referred to as “Shape-dependent region”.

In the saturation region, along with the strong absorption capability of perovskite material (high extinction coefficient value), maximized light trapping effect can be easily achieved for half apex angle decreasing from 90 to 45 degrees and remain saturated for even sharper NCs. In fact, in^4^ Lin et. al. predicted that absorption would be fully saturated even for 150 nm thick planar PTF for *λ* < 500 nm. Their prediction would justify our results, considering they had included a few layers of anti-reflective organic thin films on top of the PTF acting as the minimum light trapping nanostructures in our study.

**Figure 4:**
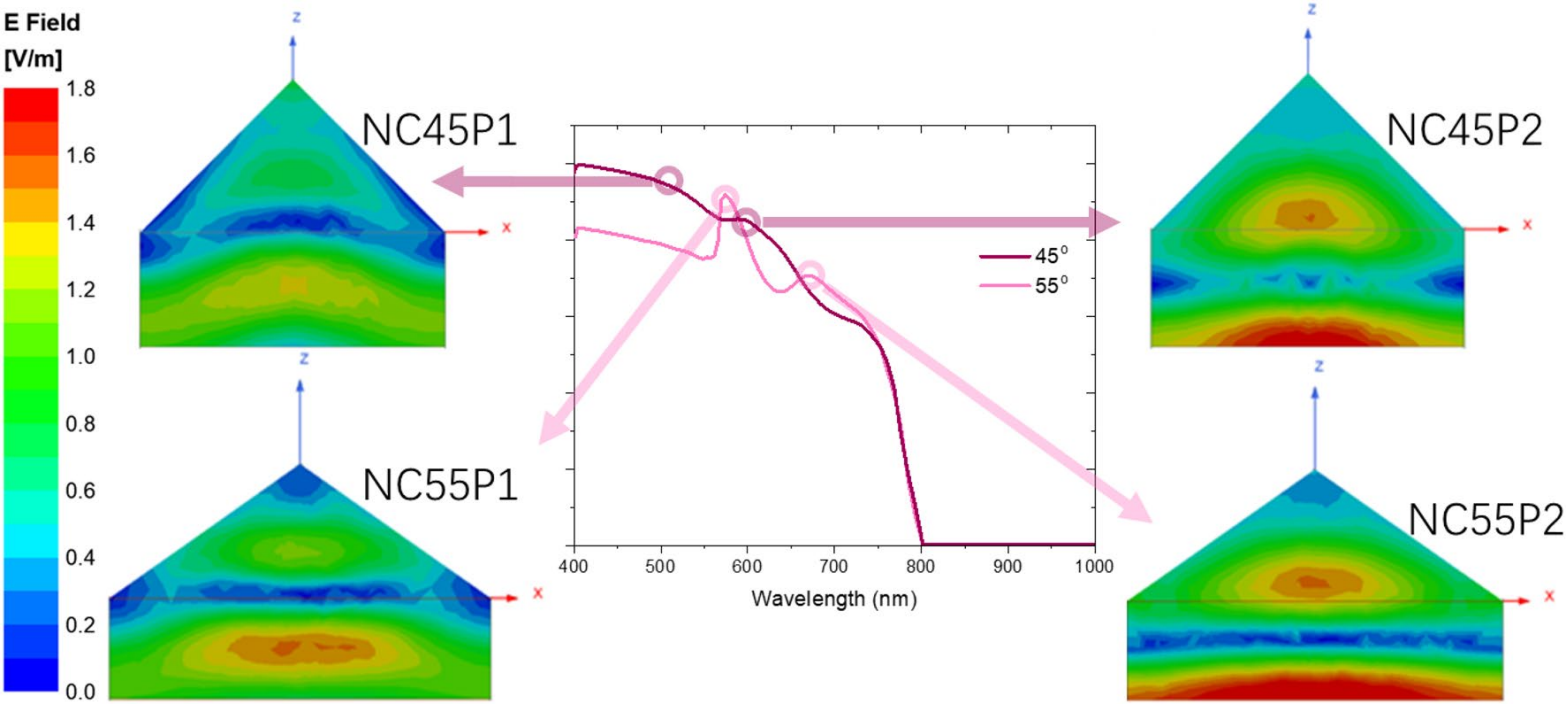
Cross sectional view of E field distribution within PTFs of NC45 and NC55.

In the shape-dependent region, the enhancement capability is strongly dependent on the apex angle. From NC25 to NC45, light trapping capability enhances monotonically. While from NC55 onwards, the light trapping capability diminishes quickly and approaching planar performance. We would argue that the improved and optimized behavior arises from below two observations. Firstly, unlike the simple destructive and constructive interferences in planar PTF governed by (2), introduction of NCs into the system would introduce extra inference modes^42^. For example, clear absorption peaks at 574 and 672 nm of NC55 design are observed. Such peaks are likely due to the incident light beam being redistributed in PTF after interaction with the nanostructure, leading to absorption “hot spots”, as shown in Fig. 4. Effectively, elongated light propagation length lead to better absorption^43^. Secondly, due to the different apex angles, even for the same mode, they would appear at a shifted wavelength. For example, in Fig. 4, distinctive interference modes can be easily identified from the E field distribution. We selected above observed two absorption peaks based on NC55 at 574 nm (NC55 P1) and 672 nm (NC55 P2). Cross compared with NC45, the same mode appeared at 522 nm (NC45 P1) and 597 nm (NC45 P2). The NC45 P1 was embedded in the saturation region and the NC45 P2’s absorption peak could be clearly observed as a shoulder peak. From second observation, as a rule of thumb, we could summarize that the larger the circular radius the longer the wavelength for the correspondent enhancement mode. We would suggest that the reason that NC25 PTF performs poorly in light trapping mainly due to interference modes exist only in saturation region and none in shape-dependent region. Similarly, NC65 PTF performs poorly mainly due to interference modes red shifts further away from shape-dependent regions. Qualitatively, we would conclude that the reason for the optimized design for NC45 and NC55 compared to the others is due to appropriate placement of the enhancement mode at the most needed wavelength range. Traditionally, the improvement of the NC design might be due to less reflection from more gradual change in refractive index^25^. Our argument of the optimized design based on mode placement would rephrase above conclusion in a different perspective. Similar to^4,44^, their planar device design would be critically dependent on the thickness of the film.

### Photovoltaic figure of merits enhancement for NC PSCs

**Figure 5:**
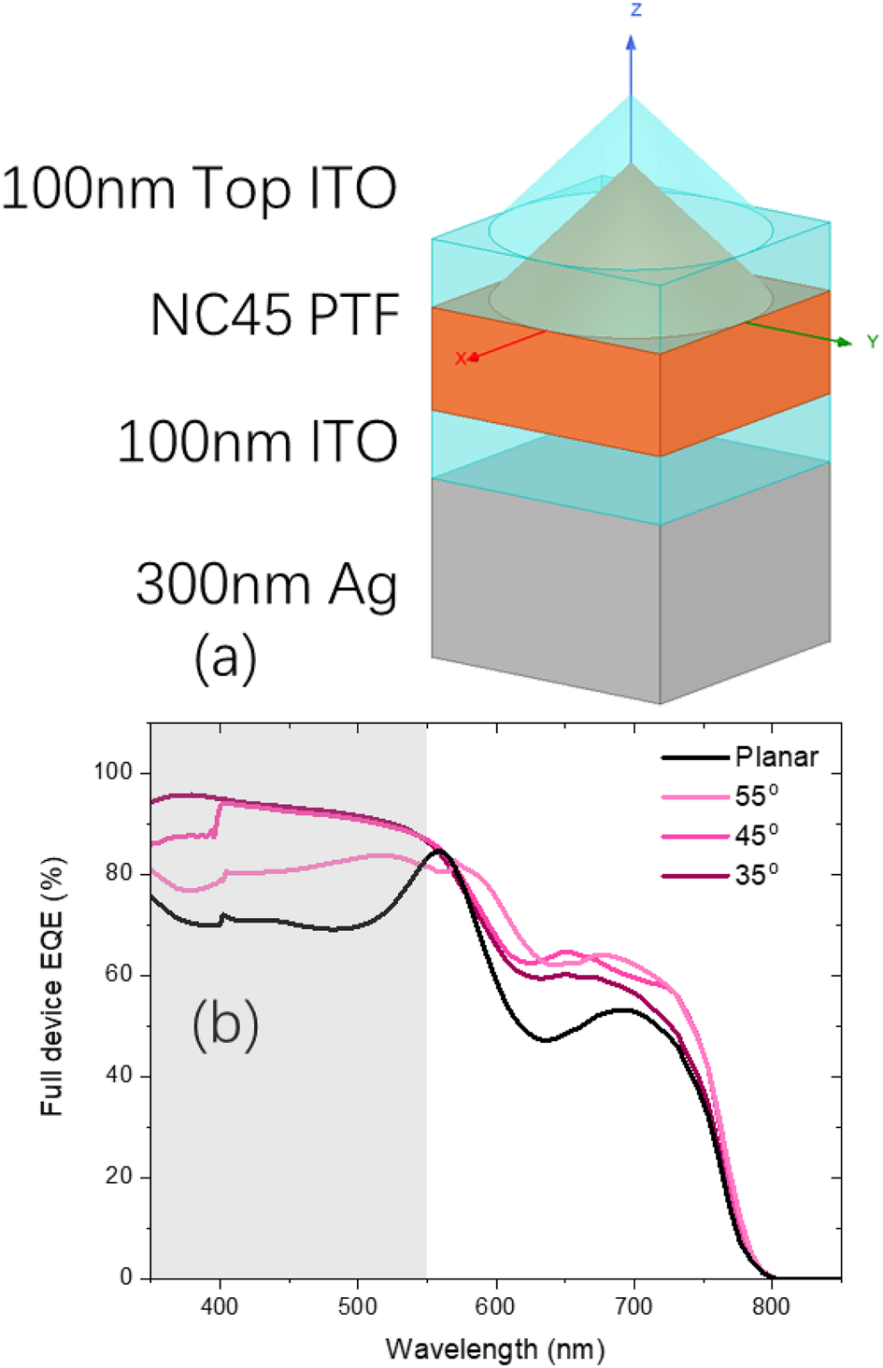
(**a**) The simulated full structure of a NC45 device unit cell; (**b**) the simulated EQE for the planar, NC35, NC45 and NC55 full devices.

**Figure 6:**
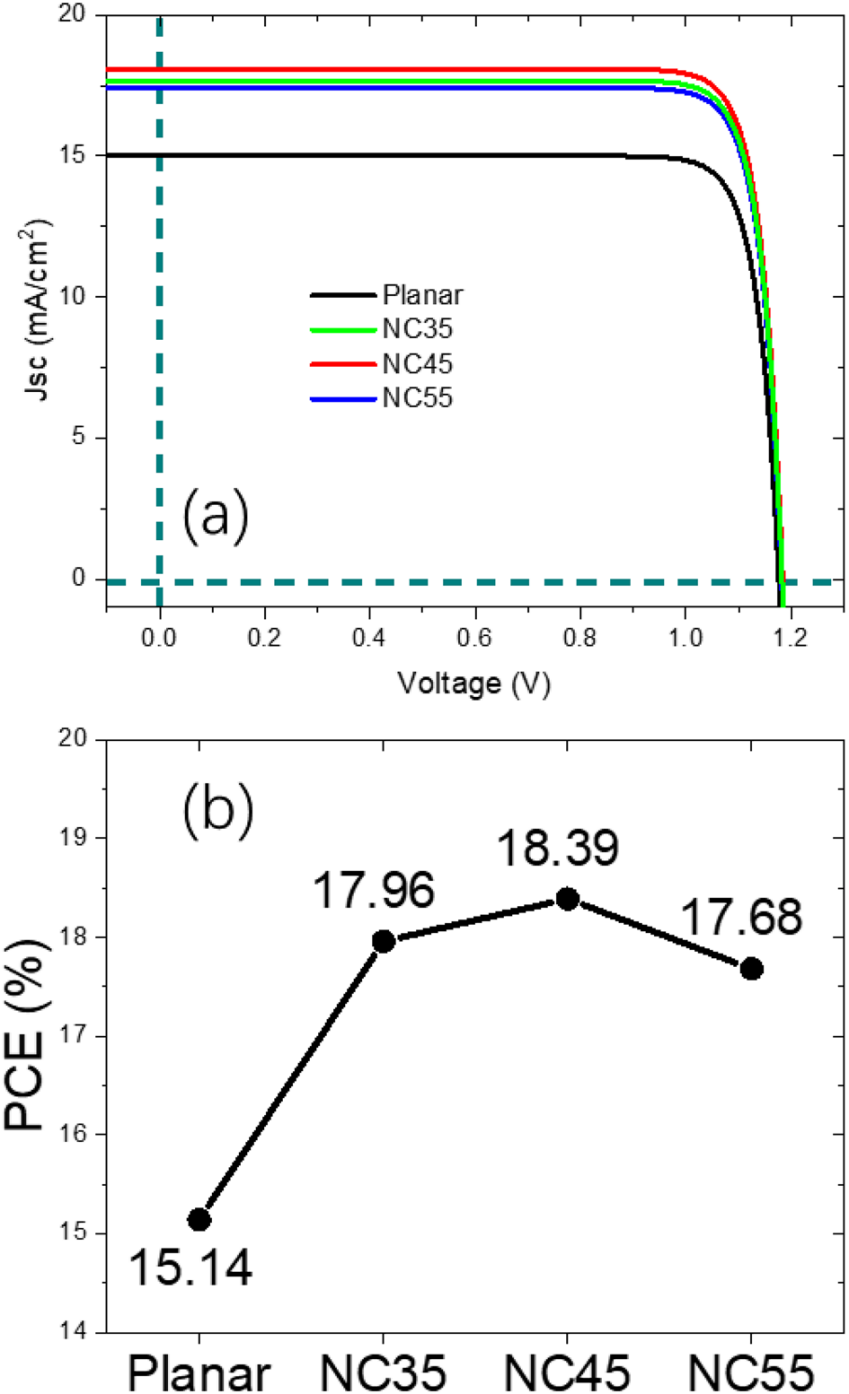
Simulated *J*–*V* curve (**a**) and PCEs (**b**) for planar, NC35, NC45 and NC55 full devices.

In the second part of the results and discussion, we further investigated the PCE performance quantitatively on the full device scale. Figure 5a shows the simulated full structure of a NC45 PV device unit cell. With referring to a typical PTF device^45^, 100 nm of ITO top contact (parameter from^46^), 100 nm of ITO as optical and electrical spacer on a 500 nm Ag back reflector (parameter from^47^) was inserted into the model. By assuming 100% internal quantum efficiency (IQE), verified by^4^, Fig. 5b shows the simulated EQE for the planar, NC35, NC45 and NC55 full devices. Compared with the absorptance spectrum in fib3b, the EQE shown has a slightly reduced performance from 5 to 10%. On one hand, the ITO/Ag back reflector directed all transmitted light back to the absorbing layer, creating a doubled or even multiplied light propagation length which favors the absorption^48^. On the other hand, parasitic absorptions of top ITO and ITO/Ag back reflectors brought down the performance^37^. Similar to light trapping capability investigation, distinctive behavior before and after *λ* at 550 nm was also observed. Further on, we calculated the *J*_*sc*_ from EQE integration (courtesy to SolarPVsoft App) and substituted *J*_*sc*_ into the ideal solar cell equation in (3) and plotted the *J-V* curve shown in Fig. 6a, where ideally factor n was set at 1.5 and open-circuit voltage (*V*_*oc*_) was set at 1.18 by *J*_*0*_ fine tuning^49^.

3 $$J = J_{sc} - J_{0} \times \exp \left[ {\left( {\frac{qV}{{nkT}}} \right) - 1} \right]$$

From Fig. 6a, a fill factor of 86% was extracted, experimentally plausible with referring to literature^2,3^. A PCE comparison as a function of the geometry design is shown in Fig. 6b. Due to the best *J*_*sc*_ performance from the optimized light trapping design, NC45 full device outperformed other devices with different apex angles. We further break down the *J*_*sc*_ into the saturation region and size-dependent region. The enhancement percentage over planar reference (Δ%) is calculated and shown in Table 2. As expected, due to the previously discussed reasons, the optimized NC45 design not only offered saturated absorption for saturation region, but also retained the uncompromised performance for size-dependent region. Unlike the previous computational studies where majority of the attention was draw on examining new types of PTF materials and defect density’s impact on the PV figure of merits of the device^26–31^, our work focuses more on the unique optical properties of PTF’s NC based nanostructures and exploring its PCE enhancement potential.

**Table 2:** *J*_*sc*_ enhancement percentage breakdown and comparison.

Structure	Total Jsc/Δ%	Saturation region/Δ%	Size-dependent region/Δ%
Planar	15.00/–	4.87/–	10.14/–
NC35	17.68/17.8	**6.36/30.6**	11.32/11.6
NC45	**18.09/20.5**	**6.22/27.8**	**11.86/17.0**
NC55	17.42/16.1	5.50/13.0	**11.92/17.5**

Significant values are in bold.

### Cross reference comparison and angle of incident robustness

**Figure 7:**
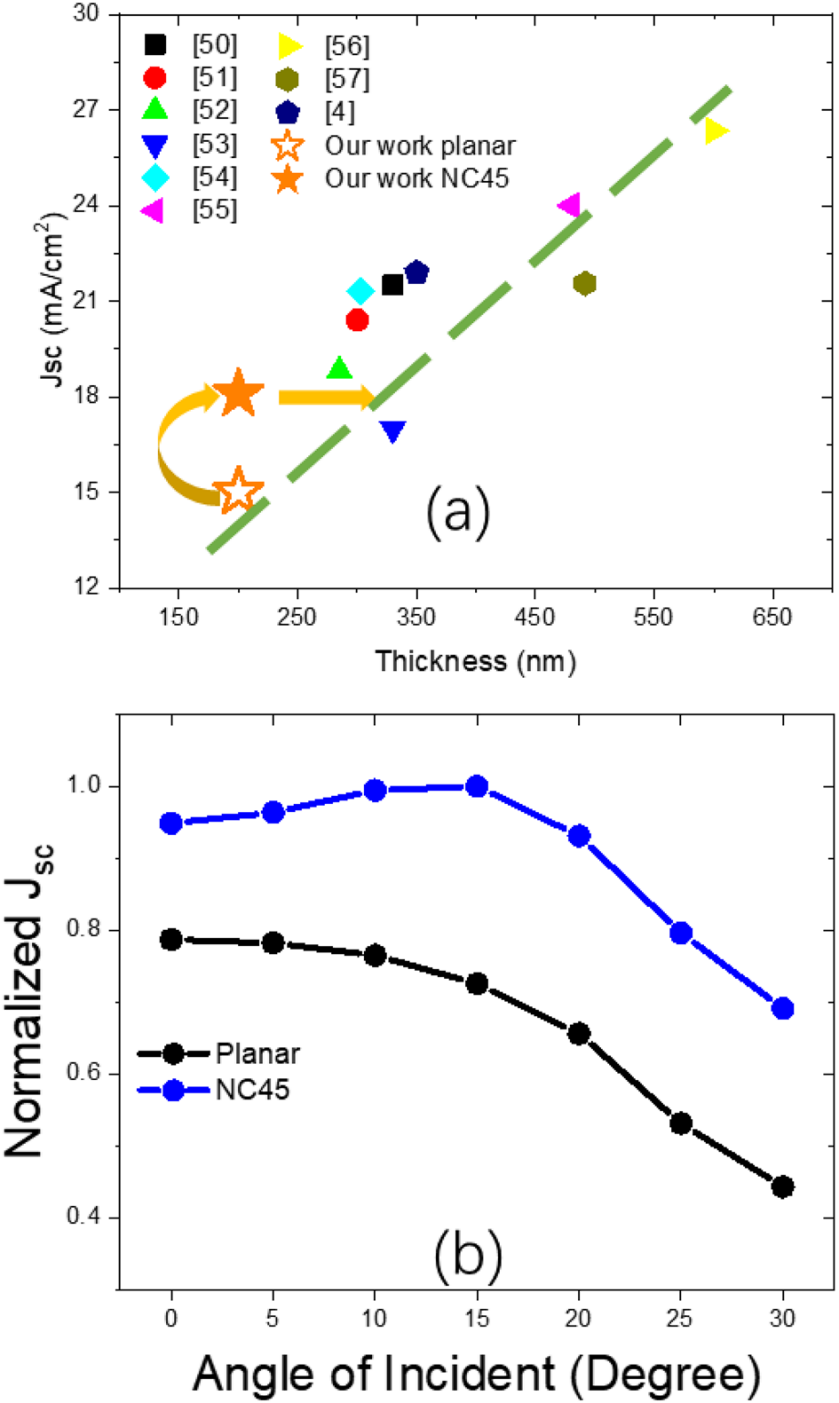
(**a**) Jsc cross comparison of our work with literature; (**b**) normalized Jsc over AOI of planar and NC45 full device.

We further compared our results with literature^4,50–57^ and plotted the Jsc versus the device thickness, as shown in Fig. 7a. Our work would suggest that with the nanostructures implementation, the performance would be equivalent to a 300 nm thick perovskite device and yet material used remain the same to a 200 nm thick case. To further prove the light trapping effectiveness of the design, we also investigated the angular dependency on full device level. Figure 7b shows the normalized *J*_*sc*_ over different angle of incident (AOI) of both planar and NC45 device. We observed a linear decreasing trend over increasing AOI for planar device, while NC45 device obtained an even higher *J*_*sc*_ at AOI = 15°. We would suggest that with an increasing AOI, higher fraction of light might had been modulated into the non-PTF layers due to wave guiding mode^9,20^. While in our design, arising from effective light trapping capabilities, the NC45 device were more robust and less sensitive to varying incident angles. This could potentially conserve additional cost for daylight tracker device in a PV micro grid.

## Conclusion

In summary, the 2D photonic nanostructured NC PTF solar cells have been designed and simulated. Excellent light trapping ability of the device has been demonstrated. Amongst NCs with different apex angle, the 45 degrees half apex angle NC structure outperforms the others. The optimized apex angle of the NCs is due to the balance between the saturated absorption for *λ* < 550 nm and optimized position tuning of the interference mode for *λ* > 550 nm. In result, we predicted a highest *J*_*sc*_ of 18.09 mA/cm^2^ for the NC45 PTF solar cell, which is 20.5% improved compared to the planar device. The *J*_*sc*_ performance is equivalently to a 300 nm thick planar PSC but with same material used. The above simulated structure could be implemented by large area nanoimprint based Anode aluminum oxide (AAO) lithography. Our work presents a significant breakthrough in PTF solar cell technology by introducing an optimized NC structure that leads to enhanced light absorption and increased efficiency. This advancement not only offers a pathway to more efficient solar cells but also underscores the potential of nanostructuring in photovoltaic technology.

## Data Availability

The datasets used and/or analysed during the current study available from the corresponding author on reasonable request.
